# Supplementary material for: Ultrasound and ultraviolet: crypsis in gliding mammals
Source: PeerJ. 2024 Mar 25;12:e17048. doi: 10.7717/peerj.17048 (PMC10977092; doi:10.7717/peerj.17048)
Supplement: Supplemental Information S1 — Details of captive sugar glider, captive springhare, and free-ranging marsupial recordings and results. Additional methods for ultraviolet-induced photoluminescence photography. [file peerj-12-17048-s001.docx]

**Supplemental Information**

**Methods**

**Captive Sugar Gliders**

**Recordings** We recorded a group of captive sugar gliders (*Petaurus breviceps*) from March 9-11^th^, 2021, in Oshawa, Ontario. The group was composed of 20 breeding pairs housed in separate enclosures within the same room; 3 pups were also present at the time of recording (a young male and female with one breeding pair and an older female with another pair). The group is cared for by a breeder (adhering to the Toronto Municipal Code (City of Toronto 2023)) who provided food and water during the recording period. We suspended an omnidirectional SMM-U1 ultrasonic microphone (Wildlife Acoustics) above the enclosures approximately 1-4m from the end of the microphone. We connected the microphone to a Song Meter SM4BAT FS ultrasonic recorder (gain = 12dB, sampling rate = 192kHz, 16-bit resolution, frequency response = 10-140kHz; Wildlife Acoustics); we set the recorder to be armed 24hrs/day and to a minimum trigger frequency of 6kHz over a 3sec window. Our recordings were non-invasive, aligning with the Animals Research Act (Government of Ontario 1990) and operating as an extension of Trent University animal care protocol 25873.

**Analyses** The resulting WAV files (10, 871 total) were analyzed using Avisoft SASLab Pro (Specht 2017). Because of the large volume of files produced, we first classified sounds manually in a small subset of files; we then validated these sound classes and used them to classify the remaining files automatically. For the initial manual classification, 432 recordings were selected from low (1900hrs = 49 files), medium (1700hrs = 82 files, 2000hrs = 65 files, 2100hrs = 66 files), and high (2200hrs = 170 files) activity hours: the sugar gliders were housed in the same room as the building’s central air unit, and therefore there was a constant low-amplitude broadband noise between 7.5 and 23.2kHz (dominant frequency = 20.2kHz) with a harmonic between 38.2 and 45.7kHz (dominant harmonic frequency = 42.7kHz) that was continuously recorded by the recording unit resulting in many otherwise empty files. Therefore, we defined activity levels by the proportion of WAV files with sounds detected by Avisoft relative to ambient noise. Low activity hours had <25% WAV files with detectable sounds, high activity hours had >75%, and medium activity levels were between 25% and 75%. We used Avisoft SAS Lab Pro to identify and manually measure sound events. This initial dataset contained 2686 sound events, which we manually classified into three categories: vocalizations, non-vocal animal sounds (sniffing and eating), and environmental noises (wheel, cage noises, unknown). We further categorized vocalizations as bark, breathing, broadband-burst, chirp, high-frequency, ultrasonic, and whistle based on key element differences (duration, peak frequency, etc.; Fig. S1). We selected representative calls from each category for templates in the remaining steps.

**Captive Springhares**

Two springhares (*Pedetes capensis*) were non-invasively recorded at the Prague Zoo over 13 days from January 31^st^ to February 27^th^, 2013. They were housed with a group of Senegal bush babies (*Galago senegalensis*), the target acoustic species for a previous study (Schneiderová et al., 2016). Both species were recorded with a Marantz PMD 662 recorder (sampling rate = 44.1kHz) protected in wire mesh. An observer would simultaneously observe the animals from the visitor’s area, and calls of springhares could be easily identified as the animals occasionally produced them when the researchers entered their enclosure to set up the recorder. We manually identified springhare vocalizations (FFT = 246, resolution = 16Hz), and characteristics were automatically measured in Avisoft SAS Lab Pro (Specht 2017). During our work, we adhered to the “Guidelines for the treatment of animals in behavioural research and teaching” (Morgan and Tromborg 2007) and the laws of the Czech Republic, the country where the research was conducted.

**Free-Ranging Marsupials**

Representative recordings of free-ranging marsupials (*Petarus australis, P. breviceps, P. norfolcensis,* and *Pseudocheirus peregrinus*) calls were provided by M. Anderson from Wild Ambience (Anderson 2022). Recordings were made using two Audio Technica AT4022 omnidirectional microphones attached to a Fostex FR2-LE Field Recorder (sampling rate = 44.1kHz), which was opportunistically deployed in various regions of Australia. *Petaurus australis* and *P. norfolcensis* were recorded in Benarkin State Forest, *P. australis* was also recorded at Twelve Mile Camp. *Petaurus breviceps* and *Pseudocheirus peregrinus* were recorded in Dunn’s Swamp in Wollemi State Park. *Petaurus norfolcensis* were recorded in the September 2018 and the other species were recorded in November 2018. Anderson non-invasively records animals by using drop rigs that are left out overnight over several recording days; he opportunistically takes only the clearest calls corresponding to animals being closer to the rig. All of Anderson’s recordings adhere to the 2014 Australian Nature Conservation Act (Australian Capital Territory 2022). Calls were manually identified (FFT = 1024, Hamming window, frame size 75%, overalp 75%) and manually measured in Avisoft SAS Lab Pro (Specht 2017).

**Photoshop Methods**

Images captured under ultraviolet (UV) conditions with the gel filter were colour-corrected to reduce the impact of visible light caused by the UV flashlight (blue-purple) and tinted gel filter (yellow). However, the combined effect was an increase in artificial cyan colouration that was not visible in normal UV conditions and was an artifact of the photo methodology. To remove this superficial cyan, we used Photoshop (Adobe Inc. 2019) to manually colour-correct the photos; we developed a simple method that was replicable with over 100 pictures in this publication and could be implemented as a standard in future studies focused on macro-analyses on UVP (i.e., without spectroscopy or multispectral imaging (Toussaint et al. 2023)):

1. Remove Cyan: We used the Hue/Saturation function to remove all cyan by selecting ‘Cyans’ from the Default menu and sliding the Saturation bar to the far left.*
2. White Balance: The resulting image from step one has an over-saturation of greys. We used the Levels function to manually correct this by selecting the grey dropper and clicking on the gray colour card that we placed in all of the photographs (included on the scale bar, printable at: <https://smallpond.ca/jim/scale/>).**

*We acknowledge that this also removes some blue from species exhibiting blue/green fluorescence under normal conditions; however, species with strong blue/green photoluminescence still retain green colouration in the final image (see *Sicista subtilis*). Therefore, the resulting four photographs (white light, UV, UV + yellow, edited) should be considered as a set to minimize the bias of other visible colours generated in this process.

**Results**

**Captive Sugar Gliders**

We recorded 275 vocalizations over three recording days; we identified six novel vocalization types (Fig. S1, Table S3), one of which was purely ultrasonic (peak frequency (kHz; ±SE) = 37.15 (±2.76); Fig. S1D). Barks (16.01 (±1.77); Fig. S1A) and broadband calls (23.74 (±4.73); Fig. S1B) also partially extended into the ultrasonic range while high-frequency calls (19.02 (±2.33); Fig. S1C), whistles (15.30; Fig. S1E), and sniffing (13.10 (±2.12); Fig. S1F) were sonic calls. Given that the ultrasonic microphones can distort calls under 20kHz, the data for high-frequency calls, whistles, and sniffing may not reflect reality and therefore, their true frequency ranges should be confirmed with sonic microphones. Additionally, the minimum frequency for barks and broadband bursts should be confirmed with sonic microphones. We did not include ultrasonic calls in further analyses as we could not remove pups from the recording space and many mammalian young can produce ultrasonic isolation calls that are lost, or modified, later in life. However, the high amplitude of barks and broadband suggest that these were produced by adults that were not confined to the nests as the pups were (hence, likely resulting in the very soft amplitudes of the ultrasonic calls).

**Captive Springhares**

We identified 105 vocalizations produced by the pair of springhares (Table S4). All calls were similar in duration (1.32s (±0.47)) and peak frequency (0.19 (±0.04)); we assigned this call type as ‘growl’ (Fig. S2E). Springhares were observed producing growls during mild arousal and growls were often paired with stomping of their hindlimbs.

**Free-Ranging Marsupials**

We identified 8 novel call types across these four species (Table S4), including yap vocalization in the sugar glider (*Petaurus breviceps*), which was not detected using ultrasonic recording equipment with captive individuals (Fig. S2B; Table S3). All calls described have low peak frequencies (<3kHz) and vary from the relatively short belch produced by *Petaurus norfolcensis* (0.16s (±0.04), Fig. S2A.3) to the long cry of *Petaurus australis* (4.02s (±0.51), Fig. SD.1&2).
