# Supplementary material for: Ultrasound and ultraviolet: crypsis in gliding mammals
Source: PeerJ. 2024 Mar 25;12:e17048. doi: 10.7717/peerj.17048 (PMC10977092; doi:10.7717/peerj.17048)
Supplement: Table S1 — Summary of references for the frequency ranges (minimum (kHz; the minimum frequency of the dominant harmonic) –maximum (kHz; the maximum frequency of the dominant harmonic)) from novel species (n = 4; methods and complete results in Info. S1) and literature (n = 70), presence of ultraviolet-induced photoluminescence (UVP; colours provided when possible) from our novel investigation (n = 64; methods provided in Info. S1 (Incon. = Inconclusive, sample sizes provided in brackets)) and previous literature (n = 19), and ecological traits (body mass (g), sociality (social: stable or cyclical social structures of multiple individuals (colonial, congregated single-burrow systems, monogamous, etc.); solitary: stable solitary lifestyle), diel activity patten (Diel A.P.; diurnal: active during the day; nocturnal: active during the night), and habitat openness (openness of dominant habitat type; closed: closed canopy, taller vegetation; open: open canopy, shorter vegetation) of gliding mammals (n = 15) and their close relatives (n = 78). Bolded species indicate those that were opportunistically sampled from museum collections. [file peerj-12-17048-s002.docx]

**Table S1. Summary of references used for the frequency range, ultraviolet-induced photoluminescence, and other traits.** Summary of references for the frequency ranges (minimum (kHz; the minimum frequency of the dominant harmonic) – maximum (kHz; the maximum frequency of the dominant harmonic)) from novel species (n = 4; methods and complete results in Info. S1) and literature (n = 69), presence of ultraviolet-induced photoluminescence (UVP; colours provided when possible) from our novel investigation (n = 57; methods provided in Info. S1 (Incon. = Inconclusive, sample sizes provided in brackets)) and previous literature (n = 25), and ecological traits (body mass (g), sociality (social: stable or cyclical social structures of multiple individuals (colonial, congregated single-burrow systems, monogamous, etc.); solitary: stable solitary lifestyle), diel activity patten (Diel A.P.; diurnal: active during the day; nocturnal: active during the night), and habitat openness (openness of dominant habitat type; closed: closed canopy, taller vegetation; open: open canopy, shorter vegetation) of gliding mammals (n = 15) and their close relatives (n = 77). Bolded species indicate those that were opportunistically sampled from museum collections.

| Species | Reference | Min–Max  Freq | UVP | Body  Mass (g) | | Gliding | Sociality | Diel  A.P. | Habitat Openness |
| --- | --- | --- | --- | --- | --- | --- | --- | --- | --- |
| **Marsupials** | | | | | | | | | |
| *Acrobates pygmaeus* | (Martin 2019) | 0.9-39 |  | 13 | | Yes | Social | Nocturnal | Closed |
|  | (Reinhold 2023) |  | Pink |  | |  |  |  |  |
| *Ailurops ursinus* | (Bool et al. 2021) | 0.19-13.5 |  | 7000 | | No | Social | Diurnal | Closed |
| *Bettongia leuseur* |  |  | No (1)^1^ | 1100 | | No | Social | Nocturnal | Open |
| *Dactylopsila trivirgata* | (Volodin 2002) | 2-3.5 |  | 407.5 | | No | Social | Nocturnal | Closed |
|  | (Reinhold 2021) |  | Blue/White |  | |  |  |  |  |
|  | (Reinhold 2023) |  | Blue/White |  | |  |  |  |  |
| *Dasyurus geoffroyi* |  |  | White (1)^1^ | 1100 | | No | Solitary | Nocturnal | Closed |
| *Dasyrus hallucatus* | (Dempster 1994) | 0.4-2 |  | 580 | | No | Solitary | Nocturnal | Closed |
|  | (Reinhold 2023) |  | Pink/  Orange/ Yellow/ Green |  | |  |  |  |  |
| ***Didelphis virginiana*** |  |  | Pink/Blue (2)^1^ | | 3950 | No | Solitary | Nocturnal | Closed |
|  | (Meisner 1983) |  | Yes |  | |  |  |  |  |
|  | (Pine et al. 1985) |  | No |  | |  |  |  |  |
|  | (Tumlison and Tumlison 2021) |  | Pink/Blue |  | |  |  |  |  |
| Species | Reference | Min–Max  Freq | UVP | Body  Mass (g) | | Gliding | Sociality | Diel  A.P. | Habitat Openness |
| **Marsupials (Continued)** | | | | | | | | | |
| ***Marmosa mexicana*** |  |  | Pink (1)^1^ | 67 | | No | Solitary | Nocturnal | Closed |
|  | (Pine et al. 1985) |  | Orange |  | |  |  |  |  |
| ***Marmosa murina*** |  |  | Pink (1)^1^ | 53.86^2^ | | No | Solitary | Nocturnal | Closed |
|  | (Toussaint et al. 2023) |  | Pink/Red |  | |  |  |  |  |
| ***Micoureus demerarae*** |  |  | Pink (1)^1^ | 108.21 | | No | Solitary | Nocturnal | Closed |
| *Perameles nasuta* | (Reinhold 2021) |  | Pink | 801.5^2^ | | No | Solitary | Nocturnal | Closed |
|  | (Reinhold 2023) |  | Pink/ Yellow/ Blue/ White |  | |  |  |  |  |
| *Petaroides volans* | (Reinhold 2023) |  | White/Grey | 1250 | | Yes | Solitary | Nocturnal | Closed |
| *Petaurus australis* | **Supp. B** | 0.25-4.63 | No (1)^1^ | 572.5^2^ | | Yes | Social | Nocturnal | Closed |
|  | (Kavanagh & Rohan-Jones 1982) | 0.7-6.5 |  |  | |  |  |  |  |
|  | (Whisson et al. 2021) | 0.5-4.5 |  |  | |  |  |  |  |
| *Petaurus breviceps* | **Supp. B** | 1.18-43.76 |  | 123.38 | | Yes | Social | Nocturnal | Closed |
| *Petaurus norfolcensis* | **Supp. B** | 0.18-3.05 |  | 245 | | Yes | Social | Nocturnal | Closed |
| *Petaurus notatus* |  |  | Incon. (1)^1^ | 128.5 | | Yes | Social | Nocturnal | Closed |
|  | (Reinhold 2021) |  | Blue/White |  | |  |  |  |  |
|  | (Reinhold 2023) |  | Pink/Blue/ White |  | |  |  |  |  |
| ***Philander opossum*** |  |  | No (1)^1^ | 437 | | No | Solitary | Nocturnal | Closed |
|  | (Pine et al. 1985) |  | Pink/ Lavender/ Yellow |  | |  |  |  |  |
| *Pseudocheirus peregrinus* | **Supp. B** | 0.46-3.25 |  | 700 | | No | Social | Nocturnal | Closed |
|  | (Reinhold 2023) |  | Green/White |  | |  |  |  |  |
| *Trichosurus vulpecula* | (Signal, Foster, & Temple 2001) | 0.5-12 | Incon. (2)^1^ | 2850 | | No | Solitary | Nocturnal | Closed |
|  | (Bolliger 1944) |  | Pink/Blue |  | |  |  |  |  |
| Species | Reference | Min–Max  Freq | UVP | Body  Mass (g) | | Gliding | Sociality | Diel  A.P. | Habitat Openness |
| **Marsupials (Continued)** | | | | | | | | | |
| *Trichosurus vulpecula* (Cont.) | (Reinhold 2023) |  | Pink/Orange/Blue |  | |  |  |  |  |
| **Primates and Relatives** | | | | | | | | | |
| ***Aotus trivirgatus*** | (Moynihan 1964) | 0.2-4.2* | No (1)^1^ | 1100^2^ | | No | Social | Nocturnal | Closed |
| ***Arctocebus calabarensis*** |  |  | White (1)^1^ | 365.5 | | No | Solitary | Nocturnal | Closed |
| *Avahi laniger* | (Stranger and Macedonia 1994) | 3.8-4 | No (1)^1^ | 950 | | No | Social | Nocturnal | Closed |
| *Callithrix jacchus* | (Epple 1968) | 0.5-15* | No (1)^1^ | 330 | | No | Social | Diurnal | Closed |
|  | (Kato et al. 2014) | 2.28-10.37 |  |  | |  |  |  |  |
|  | (Bezerra and Souto 2008) | 0.25-21.96 |  |  | |  |  |  |  |
|  | (Winter 1978) | 5.5-10.5 |  |  | |  |  |  |  |
| *Callithrix penicillata* | (Santos et al. 2017) | 5.5-10 | No (2)^1^ | 380.5 | | No | Social | Diurnal | Closed |
| *Euoticus elegantulus* |  |  | No (1)^1^ | 315 | | No | Social | Nocturnal | Closed |
| *Galago moholi* | (Anderson et al. 2000) | 0.86-2.41 |  | 196.5^2^ | | No | Social | Nocturnal | Closed |
| *Galago senegalensis* | (Zimmermann 1985)^3^ | 0.26-13.8 | No (1)^1^ | 197.5 | | No | Social | Nocturnal | Closed |
| *Galeopterus variegatus* | (Miard et al. 2019) | 37.4-39.2 | Incon. (1)^1^ | 1450 | | Yes | Solitary | Nocturnal | Closed |
| ***Lagothrix lagothricha*** | (Casamitjana 2002) | 4.45-5.41 | No (1)^1^ | 7375^2^ | | No | Social | Diurnal | Closed |
|  | (León et al. 2014)^3^ | 0.5-5.5 |  |  | |  |  |  |  |
| *Leontocebus fuscicollis* | (Moody and Menzel 1976) | 1.5-5.5 | No (1)^1^ | 332^2^ | | No | Social | Diurnal | Closed |
|  | (Zimmermann et al. 2000) | 11.7-17.5 |  |  | |  |  |  |  |
| ***Microcebus murinus*** | (Scheumann, Linn, & Zimmermann 2017) | 10-30 | Pink (1)^1^ | 60 | | No | Social | Nocturnal | Closed |
|  | (Zimmermann et al. 2000) | 12-15.6* |  |  | |  |  |  |  |
| Species | Reference | Min–Max  Freq | UVP | Body  Mass (g) | | Gliding | Sociality | Diel  A.P. | Habitat Openness |
| **Primates (Continued)** | | | | | | | | | |
| *Microcebus rufus* | (Zimmermann et al. 2000) | 14.5-32.1 |  | 50 | | No | Social | Nocturnal | Closed |
| *Microcebus sambiranensis* | (Hending et al. 2017) | 0.93-24.5 |  | 33.25 | | No | Social | Nocturnal | Closed |
| *Otolemur garnettii* | (Rosti et al. 2020)^3^ | 0.23-1.71 | Incon. (1)^1^ | 764^2^ | | No | Social | Nocturnal | Closed |
| *Paragalago cocos* | (Butynski et al. 2006) | 0.77-1.2 |  | 144^2^ | | No | Social | Nocturnal | Closed |
| *Paragalago granti* | (Butynski et al. 2006) | 0.75 |  | 150.5 | | No | Social | Nocturnal | Closed |
| *Paragalago zanzibaricus* | (Butynski et al. 2006) | 0.75-1 |  | 145 | | No | Social | Nocturnal | Closed |
| ***Plecturoebus moloch*** | (Moynihan 1966) | 0.2-7* | No (1)^1^ | 804 | | No | Social | Diurnal | Closed |
|  | (Robinson 1979) | 0.14-6.15 |  |  | |  |  |  |  |
| *Saimiri sciureus* | (Boinski and Mitchell 1995) | 0.5-2.5 | No (1)^1^ | 925 | | No | Social | Diurnal | Closed |
|  | (Boinski and Mitchell 1997) | 0.5-9.52 |  |  | |  |  |  |  |
|  | (Winter, Ploog, and Latta 1966) | 0.2-18 |  |  | |  |  |  |  |
| *Tarsius syrichta* | (Ramsier et al. 2012) | 67-79 |  | 125 | | No | Social | Nocturnal | Closed |
|  | (Řeháková-Petru et al. 2012)^3^ | 1.31-22.44 |  |  | |  |  |  |  |
| *Tarsius tarsier* | (Burton and Nietsch 2010) | 1.2-16.6 | No (1)^1^ | 116^2^ | | No | Social | Nocturnal | Closed |
|  | (Gursky 2015) | 20-80 |  |  | |  |  |  |  |
|  | (Nietsch 1999) | 3.4-16.9 |  |  | |  |  |  |  |
| *Tupaia belangeri* | (Binz and Zimmermann 1989) | 0.2-14.4 | No (1)^1^ | 150 | | No | Social | Diurnal | Closed |
| Species | Reference | Min–Max  Freq | UVP | Body  Mass (g) | | Gliding | Sociality | Diel  A.P. | Habitat Openness |
| **Rodents** | | | | | | | | | |
| *Anomalurus derbianus* |  |  | No (2)^1^ | 700 | | Yes | Social | Nocturnal | Closed |
| *Callosciurus caniceps* | (Tamura 1993) | 1.71-5.44 | No (1)^1^ | 221.1^2^ | | No | Solitary | Diurnal | Closed |
|  | (Tamura and Yong 1993) | 1.43-7.96 |  |  | |  |  |  |  |
| *Callospermophilus lateralis* | (Eiler and Banack 2004) | 4.37-10.63 | No (2)^1,3^ | 168.8^2^ | | No | Social | Diurnal | Closed |
| *Cynomys gunnisoni* | (Ackers and Slobodchikoff 1999) | 0.70-6.23 | No (2)^1,3^ | 730^2^ | | No | Social | Diurnal | Open |
|  | (Loughry et al. 2019) | 0.38-1.19 |  |  | |  |  |  |  |
|  | (Loughry, Oeser, & Hoogland 2019) | 0.39-1.18 |  |  | |  |  |  |  |
|  | (Slobodchikoff et al. 2012) | 0.5-4 |  |  | |  |  |  |  |
|  | (Perla and Slobodchikoff 2022) | 3.6-4.6 |  |  | |  |  |  |  |
|  | (Waring 1970) | 0.1-4 |  |  | |  |  |  |  |
| *Cynomys ludovicianus* | (Shannon et al. 2020) | 1.15-1.98 | Incon. (3)^1,3^ | 862^2^ | | No | Social | Diurnal | Open |
|  | (Waring 1970) | 0.1-4 |  |  | |  |  |  |  |
|  | (Wilson-Henjum et al. 2019) | 2.00-4.09 |  |  | |  |  |  |  |
| *Eliomys quercinus* | (Nummert et al. 2023) |  | Pink/Blue | 82.5 | | No | Social | Nocturnal | Closed |
| *Glaucomys sabrinus* | (Gilley 2013)^3^ | 8.12-28.72 | Pink (4)^1,3^, Blue (>10)^4^ | 141.6^2^ | | Yes | Social | Nocturnal | Closed |
|  | (Gilley et al. 2019) | 10.89-24.89 |  |  | |  |  |  |  |
|  |  |  |  |  | |  |  |  |  |
| Species | Reference | Min–Max  Freq | UVP | Body  Mass (g) | | Gliding | Sociality | Diel  A.P. | Habitat Openness |
| **Rodents (Continued)** | | | | | | | | | |
| *Glaucomys sabrinus* (Cont.) | (Murrant et al. 2013) | 20-80 |  |  | |  |  |  |  |
|  | (Kohler et al. 2019) |  | Pink/Red, Blue |  | |  |  |  |  |
| *Glaucomys volans* | (Eisinger, Scheibe, and Flaherty 2016) | 4-42 | Pink (4)^1,3^, Blue (>10)^4^ | 55.4^2^ | | Yes | Social | Nocturnal | Closed |
|  | (Gilley 2013) | 5.84-40.05 |  |  | |  |  |  |  |
|  | (Gilley et al. 2019) | 15.55-25.62 |  |  | |  |  |  |  |
|  | (Murrant et al. 2013) | 19-80 |  |  | |  |  |  |  |
|  | (Kohler et al. 2019) |  | Pink/Red, Blue |  | |  |  |  |  |
| *Hylopetes spadiceus* | (Touissant et al. 2023) |  | Pink | 65 | | Yes | Solitary | Nocturnal | Closed |
| *Ictidomys tridecemlineatus* | (Matocha 1975) | 1.40-5.93 | No (2)^1,3^ | 270 | | No | Solitary | Diurnal | Open |
|  | (Matocha 1977) | 4.16-5.92 |  |  | |  |  |  |  |
| *Marmota flaviventris* | (Blumstein and Armitage 1997) | 1-4.25* | White (2)^1^ | 3350^2^ | | No | Social | Diurnal | Open |
|  | (Davis 1991) | 2.24-4.03 |  |  | |  |  |  |  |
|  | (Matrosova et al. 2011) | 2.49-4.3 |  |  | |  |  |  |  |
|  | (Waring 1966) | 1.5-5 |  |  | |  |  |  |  |
| *Marmota vancouverensis* | (Blumstein 1999) | 1-3.8 | Incon. (1)^1^ | 6250^2^ | | No | Social | Diurnal | Open |
| *Melomys cervinipes* | (Reinhold 2021) |  | Blue/White | 72.9 | | No | Solitary | Nocturnal | Closed |
| *Mus musculus* | (Grimsley et al. 2016) | 12-64.6 | No (5)^1^ | 21 | | No | Social | Nocturnal | Open |
|  | (Hoffmann, Musolf, and Penn 2012)^3^ | 45.60-109.79 |  |  | |  |  |  |  |
| Species | Reference | Min–Max  Freq | UVP | Body  Mass (g) | | Gliding | Sociality | Diel  A.P. | Habitat Openness |
| **Rodents (Continued)** | | | | | | | | | |
| *Mus musculus* (Cont.) | (Lupanova and Egorova 2015) | 1.8-88 |  |  | |  |  |  |  |
|  | (Musolf et al. 2015)^3^ | 69.75-74.69 |  |  | |  |  |  |  |
|  | (Nyby 1983) | 70 |  |  | |  |  |  |  |
|  | (Von Merten et al. 2015)^3^ | 65.3-83.6 |  |  | |  |  |  |  |
|  | (White et al. 1998) | 40-70 |  |  | |  |  |  |  |
|  | (Zala et al. 2017)^3^ | 51.22-69.81 |  |  | |  |  |  |  |
|  | (Tumlison and Tumlison 2021) |  | No |  | |  |  |  |  |
| *Onychomys leucogaster* | (Hafner and Hafner 1979) | 10.9-11.8 | Pink (5)^1^ | 32.5 | | No | Solitary | Nocturnal | Open |
|  | (Miller and Engstrom 2012) | 10.0-11.8 |  |  | |  |  |  |  |
| *Onychomys torridus* | (Hafner and Hafner 1979) | 12.8 | Pink/White (3)^1^ | 22 | | No | Solitary | Nocturnal | Open |
| *Otospermophilus beecheyi* | (Leger, Owings, and Gelfand 1980) | 3.29-4.11 | White (2)^1,3^ | 605.1^2^ | | No | Social | Diurnal | Open |
|  | (Owings and Leger 1980) | 2-13 |  |  | |  |  |  |  |
|  | (Owings and Virginia 1978) | 2-12 |  |  | |  |  |  |  |
|  | (Rabin et al. 2003) | 3.36-6.15 |  |  | |  |  |  |  |
| *Otospermophilus variegatus* | (Krenz 1977) | 0.05-7 | White (1)^1^ | 835.5^2^ | | No | Social | Diurnal | Open |
| *Pedetes capensis* | **Supp. B** | 0.16-0.21 |  | 3500 | | No | Social | Nocturnal | Open |
|  | (Olson et al. 2021) |  | Pink/Blue |  | |  |  |  |  |
| Species | Reference | Min–Max  Freq | UVP | Body  Mass (g) | | Gliding | Sociality | Diel  A.P. | Habitat Openness |
| **Rodents (Continued)** | | | | | | | | | |
| *Peromyscus californicus* | (Miller and Engstrom 2012) | 10.8-20.2 | White (3)^1^ | 43.8 | | No | Social | Nocturnal | Closed |
|  | (Riede et al. 2022) | 11.8-81 |  |  | |  |  |  |  |
| *Peromyscus eremicus* | (Miller and Engstrom 2012) | 18.3-33.5 | Pink (3)^1^ | 25 | | No | Solitary | Nocturnal | Open |
| *Peromyscus leucopus* | (Miller and Engstrom 2012) | 18.9-27.4 | No (4)^1,3^ | 23 | | No | Solitary | Nocturnal | Closed |
| *Peromyscus maniculatus* | (Riede et al. 2022) | 6.2-79 | No (1)^1^ | 17 | | No | Social | Nocturnal | Closed |
|  | (Sales 1972) | 35-60 |  |  | |  |  |  |  |
| *Peromyscus polionotus* | (Miller and Engstrom 2012) | 20.4-27.9 | Incon. (2)^1^ | 12.5 | | No | Social | Nocturnal | Open |
| *Petaurista alborufus* | (Shen 2013)^3^ | 1.51-10.3 | No (1)^1^ | 1491.6^2^ | | Yes | Solitary | Nocturnal | Closed |
| *Petaurista leucogenys* | (Ando and Kuramochi 2008) | 0.8-12 | Incon. (1)^1^ | 1178 | | Yes | Solitary | Nocturnal | Closed |
| *Petaurista petaurista* |  |  | No (1)^1^ | 1750 | | Yes | Solitary | Nocturnal | Closed |
| *Pteromyscus pulverulentus* | (Touissant et al. 2023) |  | Pink | 193 | | Yes | Solitary | Nocturnal | Closed |
| *Rattus fuscipes* | (Barnett and Stewart 1975) | 2.5-24 |  | 130^2^ | | No | Social | Nocturnal | Closed |
|  | (Reinhold 2023) |  | Blue/White |  | |  |  |  |  |
| *Rattus rattus* | (Kaltwasser 1990) | 0.01-70 |  | 185 | | No | Social | Nocturnal | Closed |
|  | (Reinhold 2021) |  | Blue/Green |  | |  |  |  |  |
|  | (Udall et al. 1964) |  | Blue/Green |  | |  |  |  |  |
| *Reithrodontomys mexicanus* | (Miller and Engstrom 2010) | 8.24-20.12 | Pink/White (1)^1^ | 14 | | No | Solitary | Nocturnal | Closed |
| *Sciurus aberti* | (Hall 1981)^3^ | 1-4.5 | Incon. (2)^1^ | 606.2^2^ | | No | Social | Diurnal | Closed |
| *Sciurus carolinensis* | (Lishak 1982) | 0.05-14 |  | 505 | | No | Social | Diurnal | Closed |
|  | (Lishak 1984) | 0.25-16 |  |  | |  |  |  |  |
|  | (Kohler et al. 2019) |  | No |  | |  |  |  |  |
| Species | Reference | Min–Max  Freq | UVP | Body  Mass (g) | | Gliding | Sociality | Diel  A.P. | Habitat Openness |
| **Rodents (Continued)** | | | | | | | | | |
| *Sciurus carolinensis* (Cont.) | (Tumluison and Tumlison 2021) |  | No |  | |  |  |  |  |
| *Sciurus niger* | (Zelley 1971)^3^ | 0.01-5 |  | 1361 | | No | Social | Diurnal | Closed |
|  | (Kohler et al. 2019) |  | No |  | |  |  |  |  |
|  | (Tumlison and Tumlison 2021) |  | No |  | |  |  |  |  |
| *Sicista subtilis* | (Volodin et al. 2019) | 6.21-9.86 | Blue/  Green (1)^1^ | 9.1 | | No | Social | Nocturnal | Open |
| *Spermophilus citellus* | (Koshev and Pandourski 2008) | 7.1-15.33 | No (1)^1^ | 229^2^ | | No | Solitary | Diurnal | Open |
|  | (Matrosova et al. 2012) | 0.28-15.76 |  |  | |  |  |  |  |
|  | (Schneiderová 2008) | 0.13-14.81 |  |  | |  |  |  |  |
|  | (Schneiderová 2012) | 7-13 |  |  | |  |  |  |  |
|  | (Schneiderová and Policht 2012a) | 6.97-15.15 |  |  | |  |  |  |  |
|  | (Schneiderová and Policht 2012b) | 5.85-15.24 |  |  | |  |  |  |  |
|  | (Schneiderová et al. 2015) | 7.6-12 |  |  | |  |  |  |  |
|  | (Schneiderová et al. 2017) | 8.5-14 |  |  | |  |  |  |  |
|  | (Schneiderová, Štefanská, and Kratochvíl 2019) | 7.4-13.5 |  |  | |  |  |  |  |
| *Spermophilus suslicus* | (Matrosova et al. 2007) | 6.32-11.8 | White (2)^1^ | 220 | | No | Social | Diurnal | Open |
|  |  |  |  |  | |  |  |  |  |
| Species | Reference | Min–Max  Freq | UVP | Body  Mass (g) | | Gliding | Sociality | Diel  A.P. | Habitat Openness |
| **Rodents (Continued)** | | | | | | | | | |
| *Spermophilus suslicus* (Cont.) | (Matrosova et al. 2011) | 8.91-10.02 |  |  | |  |  |  |  |
|  | (Matrosova et al. 2012) | 0.18-84 |  |  | |  |  |  |  |
|  | (Matrosova et al. 2016) | 7.76-9.86 |  |  | |  |  |  |  |
|  | (Matrosova, Volodin, and Volodina 2006) | 0.01-14 |  |  | |  |  |  |  |
|  | (Matrosova, Volodin, and Volodina 2009) | 9.04-9.73 |  |  | |  |  |  |  |
|  | (Volodin 2005) | 8.97-9.76 |  |  | |  |  |  |  |
|  | (Volodin et al. 2008) | 9.29-9.70 |  |  | |  |  |  |  |
|  | (Volodina, Matrosova, and Volodin 2010) | 8.72-10.37 |  |  | |  |  |  |  |
| *Tamias amoenus* | (Brand 1976) | 1.5-11.5 | No (2)^1,3^ | 50.20^2^ | | No | Solitary | Diurnal | Open |
| *Tamias merriami* | (Brand 1976) | 1.5-15 | No (1)^1^ | 75 | | No | Social | Diurnal | Closed |
| *Tamias minimus* | (Bergstrom and Hoffmann 1991) | 1-13 | No (1)^1^ | 42.59^2^ | | No | Solitary | Diurnal | Closed |
|  | (Brand 1976) | 2-10 |  |  | |  |  |  |  |
| *Tamias ruficaudus* |  |  | No (1)^1^ | 60 | | No | Solitary | Diurnal | Closed |
| *Tamias sibiricus* | (Blake 1992) | 1.2-8.5 | No (1)^1^ | 94.85^2^ | | No | Solitary | Diurnal | Closed |
|  | (Lissovsky, Obolenskaya, and Emelyanova 2006) | 1-11.77 |  |  | |  |  |  |  |
| *Tamiasciurus douglasii* | (Smith 1978) | 0.5-8 | No (1)^1^ | 203.1^2^ | | No | Solitary | Diurnal | Closed |
|  |  |  |  |  | |  |  |  |  |
| Species | Reference | Min–Max  Freq | UVP | Body  Mass (g) | | Gliding | Sociality | Diel  A.P. | Habitat Openness |
| **Rodents (Continued)** | | | | | | | | | |
| *Tamiasciurus hudsonicus* | (Greene and Meagher 1998) | 2.4-10.1 |  | 203.5^2^ | | No | Solitary | Diurnal | Closed |
|  | (Smith 1978) | 0.5-7 |  |  | |  |  |  |  |
|  | (Kohler et al. 2019) |  | No |  | |  |  |  |  |
| *Urocitellus armatus* | (Balph and Balph 1966) | 2-7.3 | No (2)^1^ | 313 | | No | Social | Diurnal | Open |
|  | (Koeppl, Hoffmann, and Nadler 1978) | 0.05-11 |  |  | |  |  |  |  |
| *Urocitellus beldingi* | (Leger, Berney-Key, and Sherman 1984) | 3.5-5.6 | No (2)^1^ | 246.9^2^ | | No | Social | Diurnal | Open |
|  | (Robinson 1980) | 4-7 |  |  | |  |  |  |  |
|  | (Robinson 1981) | 5.9-7.1 |  |  | |  |  |  |  |
| *Urocitellus columianus* | (Betts 1976) | 0.1-10 | Incon. (3)^1,3^ | 465.7^2^ | | No | Social | Diurnal | Open |
|  | (Koeppl, Hoffmann, and Nadler 1978) | 0.01-7 |  |  | |  |  |  |  |
|  | (Manno et al. 2007) | 1.6-5.8 |  |  | |  |  |  |  |
| *Urocitellus richardsonii* | (Davis 1984) | 3.5-11 | No (1)^1^ | 667.5^2^ | | No | Social | Diurnal | Open |
|  | (Fagerstone 1987) | 0.01-8.96 |  |  | |  |  |  |  |
|  | (Koeppl, Hoffmann, and Nadler 1978) | 0.05-8.5 |  |  | |  |  |  |  |
|  | (Sloan, Wilson, and Hare 2005) | 3.18-6.31 |  |  | |  |  |  |  |
|  | (Wilson and Hare 2004) | 31.7-55 |  |  | |  |  |  |  |
|  | (Wilson and Hare 2006) | 24.4-56.3 |  |  | |  |  |  |  |
| Species | Reference | Min–Max  Freq | UVP | Body  Mass (g) | | Gliding | Sociality | Diel  A.P. | Habitat Openness |
| **Rodents (Continued)** | | | | | | | | | |
| *Urocitellus undulatus* | (Goncharov et al. 2021) | 0.05-9.8 | No (1)^1^ | 742.9 | | No | Social | Diurnal | Open |
|  | (Melchior 1971) | 1-8 |  |  | |  |  |  |  |
| *Xerospermophilus spilosoma* | (Matocha 1975)^3^ | 3.70-6.57 | No (2)^1^ | 89 | | No | Solitary | Diurnal | Open |

^1^Novel UVP observations; methods and results in Information S1
^2^Indicates sexual dimorphism; mass averaged between sexes
^3^Data taken from subspecies
^4^UVP observation confirmed with observation of live individuals in the Kawartha Highlands, ON
*Inferred from spectrograms
