# Supplementary figures and images for: Ultrasound and ultraviolet: crypsis in gliding mammals
Source: PeerJ. 2024 Mar 25;12:e17048. doi: 10.7717/peerj.17048 (PMC10977092; doi:10.7717/peerj.17048)

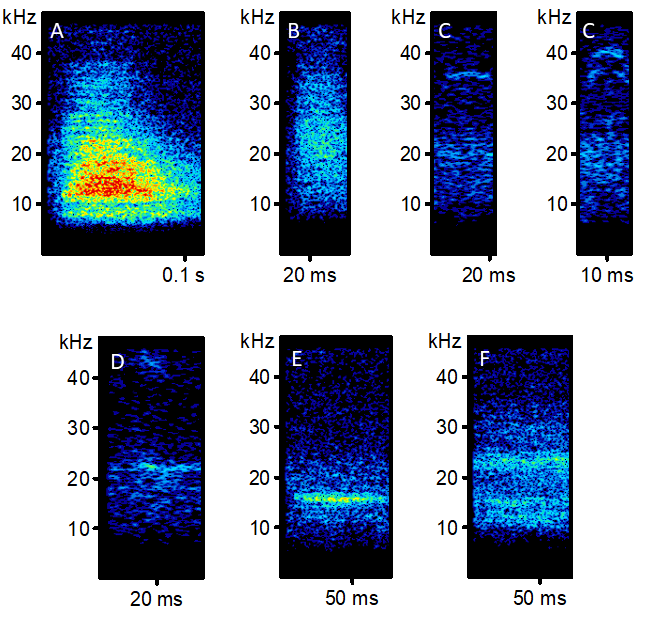

Supplement: Figure S1 — 20 breeding pairs and 3 juveniles were recorded on a Song Meter SM4BAT FS. (A) Bark (B) Broadband Burst (C) Ultrasonic (D) High Frequency (E) Whistle (F) Sniffing. [file peerj-12-17048-s003.png]

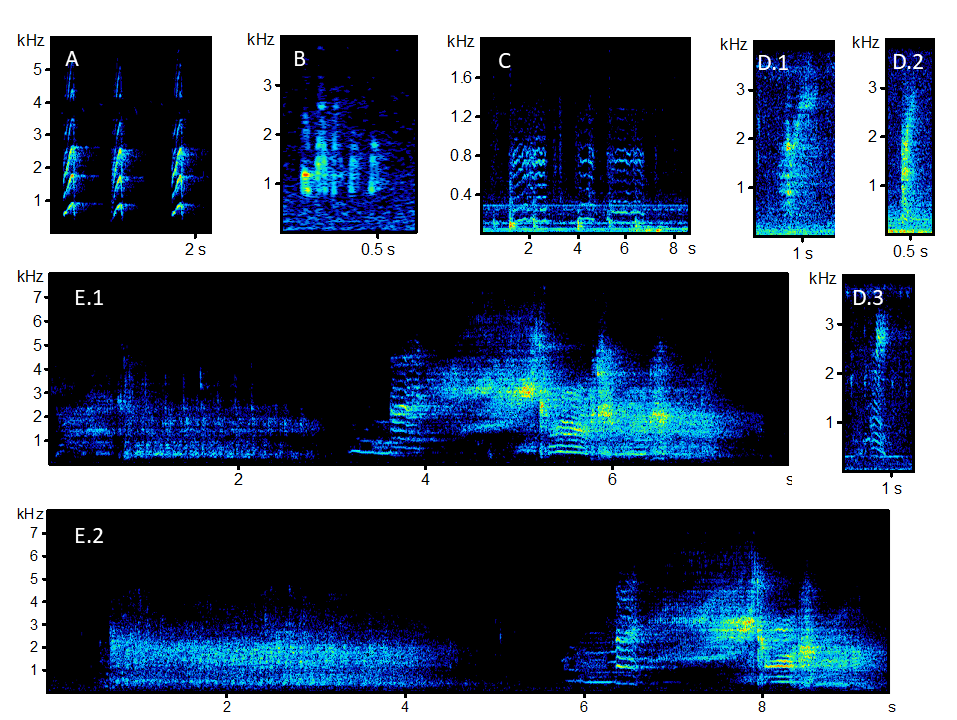

Supplement: Figure S2 — Captive springhares (Pedetes capensis) were recorded at the Prague Zoo; all other mammals are free-ranging marsupials recorded in different regions of Australia via drop-rig microphones. (A) Petaurus breviceps (Yap). (B) Pseudocheirus peregrinus (Twitter). (C) Pedetes capensis (Growl). (D) Petaurus norfolcensis (1: Nasal Grunt, 2: Belch, 3: Higher Nasal Grunt). (E) Petaurus australis (1: Gurgle and Cry, 2: Rattle and Cry). [file peerj-12-17048-s004.png]
