# Supplementary material for: Ultrasound and ultraviolet: crypsis in gliding mammals
Source: PeerJ. 2024 Mar 25;12:e17048. doi: 10.7717/peerj.17048 (PMC10977092; doi:10.7717/peerj.17048)
Supplement: Table S2 — Slope estimated ($\bar {x}$x ¯ (± SE)) are given for each variable: gliding (Y/N), diel activity pattern (Diel A.P.; nocturnal/diurnal), habitat openness (Habitat; closed/open), and sociality (solitary/social). The phylogenetic signal (s2; estimated from 1000 trees) and model fit (R2) estimations are provided. Significant effects are given in bold. [file peerj-12-17048-s005.docx]

**Table S2. Binary phylogenetic generalized linear mixed (PGLM) model results for ultraviolet-induced photoluminescence (UVP) of gliding Mammalia and their relatives (n = 83) for all reported colours (white included).** Slope estimated (*x̄* (±SE)) are given for each variable: gliding (Y/N), diel activity pattern (Diel A.P.; nocturnal/diurnal), habitat openness (Habitat; closed/open), and sociality (solitary/social). The phylogenetic signal (*s^2^*; estimated from 1000 trees) and model fit (*R^2^*) estimations are provided. Significant effects are given in bold.

|  | **Variables** | | | | | |  | **Model Fit** | |
| --- | --- | --- | --- | --- | --- | --- | --- | --- | --- |
|  | Intercept | log(Body Mass (g)) | Gliding:  *Y* | **Diel A.P.:**  ***Nocturnal*** | Habitat:  *Open* | Sociality:  *Solitary* |  | ***s^2^*** | *R^2^* |
| *x̄* (±SE) | -2.44 (±1.40) | -0.09  (±0.39) | -0.89  (±0.95) | **3.28**  **(±1.10)** | 0.76  (±0.83) | 0.36  (±0.64) |  | **0.33** | 0.51 |
| *P* | 0.08 | 0.82 | 0.35 | **0.003** | 0.36 | 0.57 |  | **0.01** |  |
