# Supplementary material for: Ultrasound and ultraviolet: crypsis in gliding mammals
Source: PeerJ. 2024 Mar 25;12:e17048. doi: 10.7717/peerj.17048 (PMC10977092; doi:10.7717/peerj.17048)
Supplement: Table S3 — Duration (s) and frequency (kHz) estimates ($\bar {x}$x ¯ (± SE)) of vocalizations produced by captive sugar gliders located in Oshawa, ON, during March 2021. Frequency estimates taken from the dominant harmonic, if harmonics present; peak frequencies represent the frequency with the highest energy. [file peerj-12-17048-s006.docx]

**Table S3. Descriptions of calls produced by captive sugar gliders (*Petaurus breviceps*) recorded with an ultrasonic microphone.** Duration (s) and frequency (kHz) estimates (x̄ (±SE)) of vocalizations produced by captive sugar gliders located in Oshawa, ON, during March 2021. Frequency estimates taken from the dominant harmonic, if harmonics present; peak frequencies represent the frequency with the highest energy.

| **Call Type** | **n** | **Duration (s)** | **Minimum (kHz)** | **Maximum (kHz)** | **Peak (kHz)** |
| --- | --- | --- | --- | --- | --- |
| Bark | 12 | 0.13 (±0.04)  [0.062-0.19] | 9.40 (±2.38)  [6.30-13.80] | 24.73 (±2.76)  [18.50-28.50] | 16.01 (±1.77)  [13.50-18.50] |
| Broadband Burst | 7 | 0.04 (±0.00)  [0.034-0.045] | 9.33 (±0.70)  [8.80-10.80] | 43.76 (± 3.96)  [35.60-47.80] | 23.74 (±4.73)  [19.10-30.50] |
| High Frequency | 5 | 0.03 (±0.00)  [0.032-0.037] | 17.62 (±2.52)  [15.00-21.00] | 20.20 (±2.51)  [17.20-23.20] | 19.02 (±2.33)  [16.50-22.10] |
| Sniffing | 2 | 0.11 (±0.05)  [0.017-0.14] | 9.50 (±0.28)  [9.30-9.70] | 16.25 (±0.78)  [15.70-16.80] | 13.10 (±2.12)  [11.60-14.60] |
| Ultrasonic | 2 | 0.02 (±0.00)  [0.016-0.022] | 33.80 (±0.14)  [33.70-33.90] | 38.15 (±3.04)  [36.00-40.30] | 37.15 (±2.76)  [35.20-39.10] |
| Whistle | 1 | 0.06 | 14.80 | 16.60 | 15.30 |
