# Supplementary material for: Ultrasound and ultraviolet: crypsis in gliding mammals
Source: PeerJ. 2024 Mar 25;12:e17048. doi: 10.7717/peerj.17048 (PMC10977092; doi:10.7717/peerj.17048)
Supplement: Table S4 — Duration (s) and frequency (kHz) estimates ($\bar {x}$x ¯ (± SE)) of vocalizations produced by captive springhares (Pedetes capensis) and various free-ranging marsupials. Frequency estimates taken from the dominant harmonic, if harmonics present; peak frequencies represent the frequency with the highest energy. [file peerj-12-17048-s007.docx]

**Table S4. Descriptions of calls from various mammals recorded with sonic microphones.** Duration (s) and frequency (kHz) estimates (x̄ (±SE)) of vocalizations produced by captive springhares *(Pedetes capensis*) and various free-ranging marsupials*.* Frequency estimates taken from the dominant harmonic, if harmonics present; peak frequencies represent the frequency with the highest energy.

| **Species** | **Call (n)** | **Duration (s)** | **Minimum (kHz)** | **Maximum (kHz)** | **Peak (kHz)** |
| --- | --- | --- | --- | --- | --- |
| *Pedetes capensis* | Growl (105) | 1.32 (±0.47)  [0.44-2.81] | 0.16 (±0.04)  [0.058-0.22] | 0.21 (±0.04)  [0.13-0.31] | 0.19 (±0.04)  [0.085-0.27] |
| *Petaurus australis* | Cry (7) | 4.02 (±0.51)  [3.33 – 4.84] | 0.25 (±0.27)  [0.09 – 0.27] | 4.63 (±0.69)  [3.37 – 5.53] | 2.07 (±0.90)  [1.21 – 3.56] |
|  | Gurgle (2) | 4.02 (±0.18)  [3.90 – 4.14] | 0.28 (±0.00) [0.28 – 0.28] | 3.60 (±0.21)  [3.46 – 3.75] | 1.77 (±0.13)  [1.68 – 1.87] |
|  | Rattle (1) | 2.48 | 0.28 | 4.21 | 0.37 |
| *Petaurus breviceps* | Yap (23) | 0.22 (±0.04) [0.13 – 0.29] | 1.18 (±0.36) [0.37 – 1.59] | 2.20 (±0.50) [0.93 – 2.71] | 2.01 (±0.51) [0.75 – 2.53] |
| *Petaurus norfolcensis* | Belch (57) | 0.16 (±0.04)  [0.021 – 0.25] | 0.18 (±0.0) [0.18 – 0.18] | 2.59 (±0.28) [1.92 – 3.51] | 1.29 (±0.59) [0.18 – 2.20] |
|  | Higher Nasal Grunt (26) | 0.19 (±0.03) [0.12 – 0.27] | 2.39 (±0.41) [0.46 – 2.62] | 3.05 (±0.045) [3.00 – 3.09] | 2.65 (±0.26) [1.40 – 2.81] |
|  | Nasal Grunt (107) | 0.49 (±0.18) [0.058 – 0.81] | 0.51 (±0.52) [0.18 – 1.78] | 2.42 (±0.23) [1.59 – 3.00] | 1.74 (±0.47) [0.18 – 2.71] |
| *Pseudocheirus peregrinus* | Twitter (9) | 0.37 (±0.05)  [0.30 – 0.43] | 0.46 (±0.13)  [0.18 – 0.65] | 3.25 (±0.56)  [2.53 – 4.50] | 1.31 (±0.32)  [0.84 – 1.78] |
