# Supplementary material for: Ultrasound and ultraviolet: crypsis in gliding mammals
Source: PeerJ. 2024 Mar 25;12:e17048. doi: 10.7717/peerj.17048 (PMC10977092; doi:10.7717/peerj.17048)
Supplement: Supplemental Information 8 — A list of all references used in the supplemental methods and results and Table S1. [file peerj-12-17048-s008.docx]

**SUPPLEMENTAL REFERENCES**

Ackers S, Slobodchikoff C. 1999. Communication of stimulus size and shape in alarm calls of Gunnison’s prairie dogs, *Cynomys gunnisoni. Ethology* 105:149–162. DOI: 10.1046/j.1439-0310.1999.00381.x.

Adobe Inc. 2019. Adobe Photoshop. Adobe Inc., San Jose, California, USA. *Available at* [*https://www.adobe.com/products/photoshop.html*](https://www.adobe.com/products/photoshop.html) (accessed 28 April 2023).

Anderson M. 2022. Wild Ambiance. *Available at* [*https://wildambience.com*](https://wildambience.com) (accessed 28 April 2023).

Anderson MJ, Ambrose L, Bearder SK, Dixson AF, Pullen S. 2000. Intraspecific variation in the vocalizations and hand pad morphology of southern lesser bush babies (*Galago moholi*): a comparison with *G. senegalensis*. *International Journal of Primatology* 21:537–555. DOI: 10.1023/A:1005400205038.

Ando M, Kuramochi Y. 2008. Vocal communication of the Japanese giant flying squirrel *Petaurista leucogenys*. *The Journal of Agricultural Science* 53:176–183.

Australian Capital Territory Government. 2022. Nature Conservation Act 2014. *Available at* [*https://www.legislation.act.gov.au/a/2014-59/default.asp*](https://www.legislation.act.gov.au/a/2014-59/default.asp) (accessed 03 August 2023).

Balph D, Balph D. 1966. Social communication of Uinta ground squirrels. *Journal of Mammalogy* 47:440–450. DOI: 10.2307/1377685.

Barnett S, Stewart A. 1975. Audible signals during intolerant behaviour of *Rattus fuscipes*. *Australian Journal of Zoology* 23:103–112. DOI: 10.1071/zo9750103.

Bergstrom B, Hoffmann R. 1991. Distribution and diagnosis of three species of chipmunks (*Tamias*) in the front range of Colorado. *The Southwestern Naturalist* 36:14–28. DOI: 10.2307/3672112.

Betts B. 1976. Behaviour in a population of Columbian ground squirrels *Spermophilus columbianus columbianus*. *Animal Behaviour* 24:652–680. DOI: 10.1016/s0003-3472(76)80079-6.

Bezerra BM, Souto A. 2008. Structure and usage of the vocal repertoire of *Callithrix jacchus*. *International Journal of Primatology* 29:671–701. DOI: 10.1007/s10764-008-9250-0.

Binz H, Zimmermann E. 1989. The vocal repertoire of adult tree shrews (*Tupaia belangeri*). *Behaviour* 109:142–162. DOI: 10.1163/156853989x00196.

Blake B. 1992. Estrous calls in captive Asian chipmunks, *Tamias sibiricus*. *Journal of Mammalogy* 73:597–603. DOI: 10.2307/1382031.

Blumstein D. 1999. Alarm calling in three species of marmots. *Behaviour* 136:731–757. DOI: 10.1163/156853999501540.

Blumstein D, Armitage K. 1997. Does sociality drive the evolution of communicative complexity? A comparative test with ground-dwelling sciurid alarm calls. *The American Naturalist* 150:179–200. DOI: 10.1086/286062.

Boinski S, Mitchell CL. 1995. Wild squirrel monkey (*Saimiri sciureus*) “caregiver” calls: contexts and acoustic structure. *American Journal of Primatology* 35:129–137. DOI: 10.1002/ajp.1350350205.

Boinski S, Mitchell CL. 1997. Chuck vocalizations of wild female squirrel monkeys (*Saimiri sciureus*) contain information on caller identity and foraging activity. *International Journal of Primatology* 18:975–993. DOI: 10.1023/A:1026300314739.

Bool I, Slaymaker M, Magrath R.D, Arfian A, Karya A, Analuddin K, Courtney Jones SK. 2021. First record of acoustic behaviour in Sulawesi bear cuscus (*Ailurops ursinus*). *Austral Ecology* 46:507–512. DOI: 10.1111/aec.12993.

Brand L. 1976. The vocal repertoire of chipmunk (genus *Eutamias*) in California. *Animal Behaviour* 24:319–335. DOI: 10.1016/s0003-3472(76)80040-1.

Burton JA, Nietsch A. 2010. Geographical variation in duet songs of Sulawesi tarsiers: evidence for new cryptic species in south and southeast Sulawesi. *International Journal of Primatology* 31:1123–1146. DOI: 10.1007/s10764-010-9449-8.

Butynski TM, De Jong YA, Perkin AW, Bearder SK, Honess PE. 2006. Taxonomy, distribution, and conservation status of three species of dwarf galagos (*Galagoides*) in eastern Africa. *Primate Conservation* 21:63–79. DOI: 10.1896/0898-6207.21.1.63.

Casamitjana J. 2002. The vocal repertoire of the woolly monkey *Lagothrix lagothricha*. *Bioacoustics* 13:1–19. DOI: 10.1080/09524622.2002.9753483.

Charlton BD. 2021. Marsupial vocal communication: a review of vocal signal production, form, and function. In: Rosenfeld CS, Hoffmann F, eds. *Neuroendocrine regulation of animal vocalization*. Academic Press; p. 297–312. DOI: 10.1016/b978-0-12-815160-0.00002-5.

City of Toronto. 2023. Toronto Municipal Code: Chapter 349, Animals. *Available at* [*https://www.toronto.ca/legdocs/municode/1184_349.pdf*](https://www.toronto.ca/legdocs/municode/1184_349.pdf) (accessed 03 August 2023).

Clink D. 2023. GibbonR: an R package for the detection and classification of acoustic signals using machine learning. *Available at* [*https://github.com/DenaJGibbon/gibbonR/*](https://github.com/DenaJGibbon/gibbonR/) (accessed 28 April 2023).

Davis L. 1984. Alarm calling in Richardon’s ground squirrels (*Spermophilus richardsonii*). *Zeitschrift für Tierpsychologie* 66:152–164. DOI: 10.1111/j.1439-0310.1984.tb01362.x.

Davis R. 1991. Semantical communication in anti-predator alarm calls. In: Hall CA, Doyle-Jones V, Widawski B, eds. *The Natural History of Eastern California and High-Altitude Research*. San Fransisco: University of California, 257–312.

Dempster ER. 1994. Vocalizations of adult Northern Quolls, *Dasyurus hallucatus*. *Australian Mammalogy* 17:43–49. DOI: 10.1071/am94005.

Eiler K, Banack S. 2004. Variability in the alarm call of golden-mantled ground squirrels *Spermophilus lateralis* and *S. saturatus*. *Journal of Mammalogy* 85:43–50. DOI: 10.1644/1545-1542(2004)085<0043:vitaco>2.0.co;2.

Eisinger J, Scheibe J, Flaherty E. 2016. Novel *Glaucomys volans* vocalizations in Indiana and evidence of geographic variation in high frequency communication. *Journal of Mammalogy* 97:1219–1227. DOI: 10.1093/jmammal/gyw076.

Epple G. 1968. Comparative studies on vocalization in marmoset monkeys (Hapalidae). *Folia Primata* 8:1–40. DOI: 10.1159/000155129.

Fagerstone K. 1987. Comparison of vocalizations between and within *Spermophilus elegans elegans* and *S. richardsonii*. *Journal of Mammalogy* 68:853–857. DOI: 10.2307/1381564.

Gilley L. 2013. Discovery and characterization of high-frequency calls in North American flying squirrels (*Glaucomys sabrinus* and *G. volans*): implications for ecology, behavior, and conservation*.* D. Phil. Thesis, Auburn University.

Gilley L, Diggins C, Pearson S, Best T. 2019. Vocal repertoire of captive northern and southern flying squirrels (*Glaucomys sabrinus* and *G. volans*). *Journal of Mammalogy* 100:518–530. DOI: 10.1093/jmammal/gyz064.

Goncharov D, Policht R, Hambálková L, Salovarov V, Hart, V. 2021. Individual-based acoustic variation of the alarm calls in the long-tailed ground squirrel. *Royal Society Open Science* 8:200147. DOI: 10.1098/rsos.200147.

Government of Ontario. 2019. Animals Research Act, R.S.O. 1990, c.A.22. *Available at* [*https://www.ontario.ca/laws/statute/90a22/v8*](https://www.ontario.ca/laws/statute/90a22/v8) (accessed 03 August 2023).

Greene E, Meagher T. 1998. Red squirrels, *Tamiasciurus hudsonicus*, produce predator-class specific alarm calls. *Animal Behaviour* 55:511–518. DOI: 10.1006/anbe.1997.0620.

Grimsley JMS., Sheth S, Vallabh N, Grimsley CA, Bhattal J, Latsko M, Jasnow A, Wenstrup JJ. 2016. Contextual modulation of vocal behavior in mouse: newly identified 12 khz “mid-frequency” vocalization emitted during restraint. *Frontiers in Behavioral Neuroscience* 10. DOI: 10.3389/fnbeh.2016.00038.

Gursky S. 2015. Ultrasonic vocalizations by the spectral tarsier, *Tarsius spectrum*. *Folia Primatologica* 86:153–163. DOI: 10.1159/000371885.

Hafner MS, Hafner DS. 1979. Vocalizations of grasshopper mice (genus *Onychomys*). *Journal of Mammalogy* 60:85–94. DOI: 10.2307/1379761.

Hall J. 1981. A field study of Kaibab squirrel in Grand Canyon National Park. *Wildlife Monographs* 75:3–54.

Hending D, Holderied M, Mccabe G. 2017. The use of vocalizations of the sambirano mouse lemur (*Microcebus sambiranensis*) in an acoustic survey of habitat preference. *International Journal of Primatology* 38:732–750. DOI: 10.1007/s10764-017-9977-6.

Hoffmann F, Musolf K, Penn DJ. 2012. Ultrasonic courtship vocalizations in wild house mice: spectrographic analyses. Journal of Ethology 30:173–180. DOI: 10.1007/s10164-011-0312-y.

Kaltwasser M-T. 1990. Acoustic signaling in the black rat (*Rattus rattus*). Journal of Comparative Psychology 104:227–232. DOI: 10.1037/0735-7036.104.3.227.

Kato Y, Gokan H, Oh-Nishi A, Suhara T, Watanabe S, Minamimoto T. 2014. Vocalizations associated with anxiety and fear in the common marmoset (*Callithrix jacchus*). Behavioural Brain Research 275:43–52. DOI: 10.1016/j.bbr.2014.08.047.

Kavanagh RP, Rohan-Jones WG. 1982. Calling behaviour of the yellow-bellied glider, *Petaurus australis* Shaw (Marsupialia: Petauridae). *Australian Mammalogy* 59:95–111. DOI: 10.1071/AM82011.

Koeppl J, Hoffmann R, Nadler C. 1978. Pattern analysis of acoustical behavior in four species of ground squirrels. *Journal of Mammalogy* 59:677–696. DOI: 10.2307/1380133.

Kohler AM, Olson ER, Martin JG, Anich PS. 2019. Ultraviolet fluorescence discovered in new world flying squirrels (*Glaucomys*). *Journal of Mammalogy* 100:21–30. DOI: 10.1093/jmammal/gyy177.

Koshev Y, Pandourski I. 2008. Structure and variability of alarm calls of European ground squirrel *Spermophilus citellus* L. 1766 (Mammalia: Rodentia) from Western Bulgaria. *Acta Zoologica Bulgarica* 60:99–105.

Krenz M. 1977. Vocalizations of the rock squirrel (*Spermophilus variegatus*). Master Thesis, Texas Tech University.

Leger D, Berney-Key S, Shermant P. 1984. Vocalizations of Belding’s ground squirrels (*Spermophilus beldingi*). *Animal Behaviour* 32:753–764.

Leger D, Owings D, Gelfand D. 1980. Single-note vocalizations of California ground squirrels: graded signals and situation-specifity of predator and socially evoked calls. *Zeitschrift für Tierpsychologie* 52:227–246. DOI: 10.1111/j.1439-0310.1980.tb00714.x.

León JJ, Vargas SA, Ramírez MA, Galvis NF, Cifuentes EF, Stevenson PR. 2014. Vocal communication in woolly monkeys (*Lagothrix lagothricha lugens*) in Cueva de los Guacharos National Park, Colombia. In: Defler TR, Stevenson PR, eds. *The woolly monkey: behavior, ecology, systematics, and captive research*. Springer, New York; p. 187–205. DOI: 10.1007/978-1-4939-0697-0_11

Lishak R. 1982. Gray squirrel mating calls: a spectrographic and ontogenic analysis. *Journal of Mammalogy* 63:661–663. DOI: 10.2307/1380274.

Lishak R. 1984. Alarm vocalizations of adult gray squirrels. *Journal of Mammalogy* 65:681–684. DOI: 10.2307/1380852.

Lissovsky A, Obolenskaya E, Emelyanova L. 2006. The structure of voice signals of Siberian chipmunk (*Tamias sibiricus* Laxmann 1769; Rodentia: Sciuridae). *Russian Journal of Theriology* 5:93–98. DOI: 10.15298/rusjtheriol.05.2.07.

Long CV. 2007. Vocalisations of the degu *Octodon degus*, a social caviomorph rodent. *Bioacoustics: The International Journal of Animal Sound and its Recording* 16:223–244. DOI: 10.1080/09524622.2007.9753579.

Loughry W, Oeser M, Anderson C, Hoogland J. 2019. The importance of individual variation in the alarm calls of Gunnison’s prairie dogs. *Animal Behaviour* 150:59–68. DOI: 10.1016/j.anbehav.2019.01.019.

Loughry W, Oeser M, Hoogland J. 2019. Alarm calls of the same individual vary during a response to the same predator in Gunnison’s prairie dogs (*Cynomys gunnisoni*). *Canadian Journal of Zoology* 97:1092–1100. DOI: 10.1139/cjz-2019-0064.

Lupanova AS, Egorova MA. 2015. Vocalization of sex partners in the house mouse (*Mus musculus*). *Journal of Evolutionary Biochemistry and Physiology* 51:324–331. DOI: 10.1134/s0022093015040080.

Manno T, Nesterova A, Debarbieri L, Kennedy S, Wright K, Dobson F. 2007. Why do male Columbian ground squirrels give a mating call? *Animal Behaviour* 74:1319–1327. DOI: 10.1016/j.anbehav.2007.02.033.

Martin K. 2019. The vocal repertoire of feathertail gliders (*Acrobates pygmaeus*) and how macrocomparisons can shape future research on acoustic communication in mammals. D. Phil. Thesis, University of New South Whales.

Matocha K. 1975. Vocal communication in ground squirrels, genus *Spermophilus.* D. Phil. Thesis, Texas Tech University.

Matocha K. 1977. The vocal repertoire of *Spermophilus tridecemlineatus*. *The American Midland Naturalist* 98:482–487. DOI: 10.2307/2424997.

Matrosova V, Blumstein D, Volodin I, Volodina E. 2011. The potential to encode sex, age, and individual identity in the alarm calls of three species of Marmotinae*. Naturwissenschaften* 98:181–192. DOI: 10.1007/s00114-010-0757-9.

Matrosova V, Rusin M, Volodina E, Proyavka S, Savinetskaya L, Shekarova O, Rashevska H, Volodin I. 2016. Genetic and alarm call diversity across scattered populations of speckled ground squirrels (*Spermophilus suslicus*). *Mammalian Biology* 81:255–265. DOI: 10.1016/j.mambio.2016.01.001.

Matrosova V, Schneiderová I, Volodin I, Volodina E. 2012. Species-specific and shared featured in vocal repertoires of three Eurasian ground squirrels (genus *Spermophilus*). *Acta Theriologica* 57:65–78. DOI: 10.1007/s13364-011-0046-9.

Matrosova V, Volodin I, Volodina E. 2006. The diversity of calls produced by live-trapped speckled ground squirrels *Spermophilus suslicus* (Rodentia, Sciuridae) (in Russian). *Bulletin of Moscow Society of Naturalists* 111:84–87.

Matrosova V, Volodin I, Volodina E. 2009. Short-term and long-term individuality in speckled ground squirrel alarm calls. *Journal of Mammalogy* 90:158–166. DOI: 10.1644/08-mamm-a-032.1.

Matrosova V, Volodin I, Volodina E, Babitsky A. 2007. Pups crying bass: vocal adaptation for avoidance of age-dependent predation risk in ground squirrels? *Behavioural Ecology and Sociobiology* 62:181–191. DOI: 10.1007/s00265-007-0452-9.

Meisner DH. 1983. Psychedelic opossums-fluorescence of the skin and fur of *Didelphis virginiana kerr*. *Ohio Journal of Science* 83:4.

Melchior H. 1971. Characteristics of arctic ground squirrel alarm calls. *Oecologia* 7:184–190. DOI: 10.1007/bf00346360.

Miard P, Lim LS, Abdullah NI, Elias NA, Ruppert N. 2019. Ultrasound use by sunda colugos offers new insights into the communication of these cryptic mammals. *Bioacoustics* 28:397–403. DOI: 10.1080/09524622.2018.1463294.

Miller JR, Engstrom MD. 2010. Stereotypic vocalizations in harvest mice (*Reithrodontomys*): harmonic structure contains prominent and distinctive audible, ultrasonic, and non-linear elements. *The Journal of the Acoustical Society of America* 128:1510. DOI: 10.1121/1.3455855.

Miller JR, Engstrom MD. 2012. Vocal stereotypy in the rodent genera *Peromyscus* and *Onychomys* (Neotominae): taxonomic signature and call design. *Bioacoustics* 21:193–213. DOI: 10.1080/09524622.2012.675176.

Moody MI, Menzel EW Jr. 1976. Vocalizations and their behavioral contexts in the tamarin *Saguinis fuscicollis. Folia Primata* 25:73–94. DOI: 10.1159/000155708.

Morgan KN, Tromborg CT. 2007. Sources of stress in captivity. *Applied Animal Behaviour Science* 102:262–302. DOI: 10.1016/j.applanim.2006.05.032.

Moynihan M. 1964. Some behavior patterns of the platyrrhine monkeys I. The night monkey (*Aotus trivirgatus*). *Smithsonian Miscellaneous Collections* 146:1–84.

Moynihan M. 1966. Communication in the Titi monkey, *Callicebus*. *Journal of Zoology* 150:77–127. DOI: 10.1111/j.1469-7998.1966.tb02999.x.

Murrant M, Bowman J, Garroway C, Prinzen B, Mayberry H, Faure P. 2013. Ultrasonic vocalizations emitted by flying squirrels. *PLOS ONE* 8:1–6. DOI: 10.1371/journal.pone.0073045.

Musolf K, Meindl S, Larsen AL, Kalcounis-Rueppell MC, Penn DJ. 2015. Ultrasonic vocalizations of male mice differ among species and females show assortative preferences for male calls. *PLOS ONE* 10:e0134123. DOI: 10.1371/journal.pone.0134123.

Nietsch A. 1999. Duet vocalizations among different populations of Sulawesi tarsiers. *International Journal of Primatology* 20:567–583.

Nummert G, Ritson K, Nemvalts K. 2023. Photoluminescence in the garden dormouse (*Eliomys quercinus*). *Zoology* 157. DOI: 10.1016/j.zool.2023.126075.

Nyby J. 1983. Ultrasonic vocalizations during sex behavior of male house mice (*Mus musculus*): a description. *Behavioral and Neural Biology* 39:128–134. DOI: 10.1016/s0163-1047(83)90722-7.

Olson ER, Carlson M, Ramanujam V, Sears L, Anthony S, Anich P, Ramon L, Hulstrand A, Jurewicz M, Gunnelson A, Kohler A, Martin J. 2021. Vivid biofluorescence discovered in the nocturnal springhare (Pedetidae). *Scientific Reports* 11:4125. DOI: 10.1038/s41598-021-83588-0.

Owings D, Leger D. Chatter vocalizations of California ground squirrels: predator- and social-role specificity*. Zeitschrift für Tierpsychologie* 54:163–184. DOI: 10.1111/j.1439-0310.1980.tb01070.x.

Owings D, Virginia R. Alarm calls of California ground squirrels (*Spermophilus beecheyi*). *Zeitschrift für Tierpsychologie* 46:58–70. DOI: 10.1111/j.1439-0310.1978.tb01438.x.

Perla BS, Slobodchikoff CN. 2002. Habitat structure and alarm call dialects in Gunnison’s prairie dog (*Cynomys gunnisoni*). *Behavioral Ecology* 13:844–850. DOI: 10.1093/beheco/13.6.844.

Pine RH, Rice JE, Bucher JE, Tank Jr DH, Greenhall AM. 1985. Labile pigments and fluorescent pelage in didelphid marsupials. *Mammalia* 49:249–256.

R Development Core Team. 2022. R: a language and environment for statistical computing. R Foundation for Statistical Computing, Vienna, Austria. *Available at* [*https://www.r-project.org/*](https://www.r-project.org/) (accessed 28 April 2023).

Rabin L, McCowan B, Hooper S, Owings D. 2003. Anthropogenic noise and its effect on animal communication: an interface between comparative psychology and conservation biology. *International Journal of Comparative Psychology* 16:172–192. DOI: 10.46867/c4f59p.

Ramsier MA, Cunningham AJ, Moritz GL, Finneran JJ, Williams CV, Ong PS, Gursky-Doyen SL, Dominy NJ. 2012. Primate communication in the pure ultrasound. *Biology Letters* 8:508–511. DOI: 10.1098/rsbl.2011.1149.

Řeháková-Petru M, Policht R, Peške L. 2012. Acoustic repertoire of the Philippine tarsier (*Tarsius syrichta fraterculus*) and individual variation of long-distance calls. *International Journal of Zoology* 2012:602401. DOI: 10.1155/2012/602401.

Reinhold L. 2021. Mammals with fluorescent fur: observations from the wet tropics. *North Queensland Naturalist* 51:1–8. DOI: 10.53060/prsq23.5.

Reinhold L. 2023. Photoluminescence in Fur. M. Phil. Thesis, James Cook University.

Riede T, Kobrina A, Bone L, Darwaiz T, Pasch B. 2022. Mechanisms of sound production in deer mice (*Peromyscus* spp.). *Journal of Experimenal Biology* 225:jeb243695. DOI: 10.1242/jeb.243695.

Robinson JG. 1979. An analysis of the organization of vocal communication in the Titi monkey *Callicebus moloch. Zeitschrift für Tierpsychologie* 49:381–405. DOI: 10.1111/j.1439-0310.1979.tb00300.x.

Robinson S. 1980. Antipredator behaviour and predator recognition in Belding’s ground squirrels. *Animal Behaviour* 28:840–852. DOI: 10.1016/s0003-3472(80)80144-8.

Robinson S. 1981. Alarm communication in Belding’s ground squirrels. *Zeitschrift für Tierpsychologie* 56:150–168. DOI: 10.1111/j.1439-0310.1981.tb01293.x.

Rosti H, Pihlström H, Bearder S, Pellikka P, Rikkinen J. 2020. Vocalization analyses of nocturnal arboreal mammals of the Taita Hills, Kenya. *Diversity* 12:1–23. DOI: 10.3390/d12120473.

Sales GD. 1972. Ultrasound and mating behaviour in rodents with some observation on other behavioural situations. *Journal of Zoology* 168:149–164. DOI: 10.1111/j.1469-7998.1972.tb01345.x.

Santos SG, Duarte MHL, Sousa-Lima RS, Young RJ. 2017. Comparing contact calling between black tufted-ear marmosets (*Callithrix penicillata*) in a noisy urban environment and in a quiet forest: noise effects on contact calls in black tufted-ear marmosets. *International Journal of Primatology* 38:1130–1137. DOI: 10.1007/s10764-017-0002-x.

Scheumann M, Linn S, Zimmermann E. 2017. Vocal greeting during mother-infant reunions in a nocturnal primate, the gray mouse lemur (*Microcebus murinus*). *Scientific Reports* 7:1–7. DOI: 10.1038/s41598-017-10417-8.

Schneiderová I. 2008. Acoustics communication and alarm calls in two species of ground squirrels *Spermophilus citellus* and *Spermophilus taurensis* (Sciuridae; Rodentia). D. Phil. Thesis, Charles University.

Schneiderová I. 2012. Frequency-modulated second elements of two-element alarm calls do not enhance discrimination of callers in three Eurasian ground squirrels. *Current Zoology* 58:749–757. DOI: 10.1093/czoolo/58.5.749.

Schneiderová I, Policht R. 2012a. Acoustic analysis of alarm calls of the European ground squirrel (*Spermophilus citellus*) and the Taurus ground squirrel (*S. taurensis*) (Mammalia: Sciuridae). *Zoologischer Anzeiger* 251:139–146. DOI: 10.1016/j.jcz.2011.07.005.

Schneiderová I, Policht R. 2012b. Acoustic analysis of the alarm calls of the Anatolian ground squirrel *Spermophilus xanthoprymnus*: a description and comparison with alarm calls of the Taurus *S. taurensis* and European *S. citellus* ground squirrels*. Naturwissenschaften* 99:55–64. DOI: 10.1007/s00114-011-0870-4.

Schneiderová I, Schnitzerová P, Uhlíková J, Brandl P, Zouhar J, Matějů J. 2015. Differences in alarm calls of juvenile and adult European ground squirrels (*Spermophilus citellus*): findings on permanently marked animals from a semi-natural enclosure*. Zoo Biology* 34:503–512. DOI: 10.1002/zoo.21233.

Schneiderová I, Štefanská L, Kratochvíl L. 2019. Geographic variability in the alarm calls of the European ground squirrel*. Current Zoology* November:1–9. DOI: 10.1093/cz/zoz055.

Schneiderová I, Volodina E, Matrosova V, Volodin I. 2017. One plus one: binary alarm calls retain individual signature for longer periods than single-note alarms in the European ground squirrel (*Spermophilus citellus*). *Behavioural Processes* 138:73–81. DOI: 10.1016/j.beproc.2017.02.014.

Schneiderová I, Zouhar J, Štefanská L, Bolfíková BČ, Lhota S, Brandl P. 2016. Vocal activity of lesser galagos (*Galago* spp.) at zoos. *Zoo Biology* 35:147–156. DOI: 10.1002/zoo.21261. DOI: 10.1002/zoo.21261.

Shannon G, McKenna M, Wilson-Henjum G, Angeloni L, Crooks K, Wittemyer G. 2020. Vocal characteristics of prairie dog alarm calls across an urban noise gradient. *Behavioral Ecology* 31:393–400. DOI: 10.1093/beheco/arz200.

Shen P. 2013. Acoustic behavior of white-faced flying squirrel (*Petaurista lena*) in Guanghua Village, Alishan. Master Thesis, National Sun Yat-sen University.

Signal T, Foster TM, Temple W. 2001. Determination of auditory thresholds in the brushtail possum (*Trichosurus vulpecula*). *Physiology & Behavior* 23:195–200. DOI: 10.1016/s0031-9384(01)00446-2.

Sloan J, Wilson D, Hare J. 2005. Functional morphology of Richardson’s ground squirrel, *Spermophilus richardsonii*, alarm calls: the meaning of chirps, whistles and chucks. *Animal Behaviour* 70:937–944. DOI: 10.1016/j.anbehav.2005.01.013.

Slobodchikoff C, Briggs W, Dennis P, Hodge A. 2012. Size and shape information serve as labels in the alarm calls of Gunnison’s prairie dogs *Cynomys gunnisoni. Current Zoology* 58:741–748. DOI: 10.1093/czoolo/58.5.741.

Smith C. 1978. Structure and function of the vocalizations of tree squirrels (*Tamiasciurus*). *Journal of Mammalogy* 59:793–808. DOI: 10.2307/1380144.

Specht R. 2017. Avisoft-SASLab Pro: sound analysis and synthesis laboratory. Avisoft Bioacoustics E.K.

Stranger KF, Macedonia JM. 1994. Vocalizations of aye-ayes (*Daubentonia madagascariensis*) in captivity. *Folia Primatologica* 62:160–169. DOI: 10.1159/000156773.

Tamura N. 1993. Role of sound communication in mating Malaysian *Callosciurus* (Sciuridae). *Journal of Mammalogy* 74:468–476. DOI: 10.2307/1382404.

Tamura N, Yong H. 1993. Vocalizations in response to predators in three species of Malaysian *Callosciurus* (Sciuridae). *Journal of Mammalogy* 74:703–714. DOI: 10.2307/1382292.

Toussaint SLD, Ponstein J, Thoury M, Métivier R, Kalthoff DC, Habermeyer B, Guilard R, Bock S, Mortensen P, Sandberg S. 2023. Fur glowing under ultraviolet: in situ analysis of porphyrin accumulation in the skin appendages of mammals. *Integrative Zoology* 18:15–26. DOI: 10.21203/rs.3.rs-499384/v3.

Tumlison R, Tumlison TL. 2021. Investigation of fluorescence in selected mammals of Arkansas. *Journal of the Arkansas Academy of Science* 75:29–35. DOI: 10.54119/jaas.2021.7515.

Udall SL, Briggs FP, Pautzke CF, Janzen DH. 1964. Fluorescence studies. In *Wildlife research: problems, programs, progress.* United States Department of the Interior, Fish and Wildlife Service and Bureau of Sport Fisheries and Wildlife, Washington; p. 64.

Volodin I. 2002. Uninterrupted vocalization in the striped possum *Dactylopsila trivirgata* (Marsupialia, Petauridae) during the whole respiratory cycle. *Zoologicheskiĭ Zhurnal* 81:1526–1529.

Volodin I. 2005. Individuality of alarm calls in the spotted suslik (*Spermophilus suslicus*, Rodentia, Sciuridae) (in Russian). *Zoological Journal* 84:228–235.

Volodin IA, Klenova AV, Ilchenko OG, Volodina EV. 2019. High frequency audible calls in northern birch mice *Sicista betulina* in response to handling: effects of individuality, sex and body mass on the acoustics*. BMC Research Notes* 12:677. DOI: 10.1186/s13104-019-4719-9.

Volodin I, Volodina E, Matrasova V, Savinetskaya L, Shekarova O, Voytsik V. 2008. Population density does not affect the alarm call characteristics in the speckled ground squirrel (*Spermophilus suslicus*). *Lynx (Praha)* 39:333–342.

Volodina E, Matrosova V, Volodin I. 2010. An unusual effect of maturation on the alarm call fundamental frequency in two species of ground squirrels. *Bioacoustics* 20:87–98. DOI: 10.1080/09524622.2011.9753634.

Von Merten S, Hoier S, Pfeifle C, Tautz D. 2015. A role for ultrasonic vocalisation in social communication and divergence of natural populations of the house mouse (*Mus musculus domesticus*). *PLOS ONE* 10:e0118130. DOI: 10.1371/journal.pone.0097244.

Waring G. 1966. Sounds and communications of the yellow-bellied marmot (*Marmota flaviventris*). *Animal Behaviour* 14:177–183. DOI: 10.1016/s0003-3472(66)80028-3.

Waring G. 1970. Sound communications of black-tailed, white-tailed, and Gunnison’s prairie dogs. *The American Midland Naturalist* 83:167–185. DOI: 10.2307/2424014.

Whisson DA, McKinnon F, Lefoe M, Rendall AR. 2021. Passive acoustic monitoring for detecting the yellow-bellied glider, a highly vocal arboreal marsupial. *PLOS ONE* 16:e0252092. DOI: 10.1371/journal.pone.0252092.

White NR, Prasad M, Barfield RJ, Nyby JG. 1998. 40- and 70-khz vocalizations of mice (*Mus musculus*) during copulation. *Physiology & Behavior* 63:467–473. DOI: 10.1016/s0031-9384(97)00484-8.

Wilson D, Hare J. 2004. Ground squirrel uses ultrasonic alarms*. Nature* 430:523. DOI: 10.1038/430523a.

Wilson D, Hare J. 2006. The adaptive utility of Richardson’s ground squirrel (*Spermophilus richardsonii*) short-range ultrasonic alarm signals. *Canadian Journal of Zoology* 84:1322–1330. DOI: 10.1139/z06-120.

Wilson-Henjum G, Job J, McKenna M, Shannon G, Wittemyer G. 2019. Alarm call modification by prairie dogs in the presence of juveniles. *Journal of Ethology* 37:167–174. DOI: 10.1007/s10164-018-0582-8.

Winter M. 1978. Some aspects of the ontogeny of vocalizations of hand-reared common marmosets. In Roth H, Wolters H-J, Hearn JP, eds*. Biology and Behaviour of Marmosets*. Göttingen: Eigenverlag Rothe, 127–139.

Winter P, Ploog D, Latta J. 1966. Vocal repertoire of the squirrel monkey (*Saimiri sciureus*), its analysis and significance. *Experimental Brain Research* 1:359–384. DOI: 10.1007/bf00237707.

Zala SM, Reitschmidt D, Noll A, Balazs P, Penn DJ. 2017. Sex-dependent modulation of ultrasonic vocalizations in house mice (*Mus musculus musculus*). *PLOS ONE* 12:e0188647. DOI: 10.1371/journal.pone.0188647.

Zelley R. The sounds of the fox squirrel, *Sciurus niger rufiventer*. *Journal of Mammalogy* 52:597–604. DOI: 10.2307/1378595.

Zimmermann E. 1985. The vocal repertoire of the adult Senegal bushbaby (*Galago senegalensis senegalensis*). *Behaviour* 94:212–233. DOI: 10.1163/156853985x00190.

Zimmermann E, Vorobieva E, Wrogemann D, Hafen T. 2000. Use of vocal fingerprinting for specific discrimination of gray (*Microcebus murinus*) and rufous mouse lemurs (*Microcebus rufus*). *International Journal of Primatology* 21:837–852.
